# An interpretable machine learning approach for predicting and grading hip osteoarthritis using gait analysis

**DOI:** 10.1186/s12891-025-08911-6

**Published:** 2025-07-01

**Authors:** Qing Yang, Xinyu Ji, Yuyan Zhang, Shaoyi Du, Bing Ji, Wei Zeng

**Affiliations:** 1 Department of Breast and Thyroid Surgery, Shandong Provincial Hospital Affiliated to Shandong First Medical University, Jing Wu Wei Qi Road, Jinan, 250021 Shandong Province People’s Republic of China; 2 School of Control Science and Engineering, Shandong University, Jingshi Road, Jinan, 250061 Shandong Province People’s Republic of China; 3 Institute of Artificial Intelligence and Robotics, Xi’an Jiaotong University, Xianning West Road, Xi’an, 710049 Shaanxi Province People’s Republic of China; 4 School of Physics and Electromechanical Engineering, Longyan University, Dongxiao North Road, Longyan, 364012 Fujian Province People’s Republic of China

**Keywords:** Hip osteoarthritis (OA), Spatiotemporal parameters, Nonlinear features, Gait analysis, Machine learning, Interpretability analysis

## Abstract

**Background:**

Osteoarthritis (OA) of the hip is a progressive musculoskeletal disorder characterized by stiffness and limited passive range of motion. Hip OA patients experience mobility impairment and altered gait patterns when compared to healthy controls (HCs). Although various interventions have been designed to alleviate these symptoms, it is unclear if there is a reliable method to track biomechanical changes in patients with unilateral hip OA in a clinical setting.

**Purpose:**

The purpose of this study is to evaluate the efficacy of lower extremity kinematic gait data for detecting and rating the severity of unilateral hip OA using machine learning algorithms.

**Methods:**

First, a feature extraction framework is developed to derive several discriminative spatiotemporal and nonlinear features from lower extremity kinematic gait data. These features reflect the subtle disparity in gait characteristics, and can serve as indicators to distinguish between groups. Afterwards, the Shapley Additive exPlanations (SHAP) method is applied for feature selection and dimensionality reduction, providing detailed explanations of each feature’s contribution to classification performance. Second, a support vector machine (SVM) is used to classify gait patterns between unilateral hip OA patients and HCs. Finally, the effectiveness of this strategy is comprehensively validated on a publicly available gait dataset, containing 80 asymptomatic participants and 99 patients with unilateral hip OA, who are classified according to Grades 2, 3, and 4 of Kellgren and Lawrence (KL).

**Results:**

Using a cross-validation scheme of 10-fold, the classification accuracy achieves 98.21% for hip OA detection (HCs *vs* hip OA patients) and 89.65% (HCs *vs* Grade2/3 *vs* Grade 4) and 87.54% (HCs *vs* Grade2 *vs* Grade 3 *vs* Grade 4) for severity rating.

**Conclusion:**

The results demonstrate superior performance compared to other up-to-date methods, suggesting that the proposed method can serve as a supplementary tool to the KL grading scale for hip OA detection and severity assessment in clinical practice. Gait analysis provides objective data on the patient’s walking pattern and can detect subtle changes in gait that may not be apparent on a radiographic image.

**Trial registration:**

ClinicalTrials. gov (NCT01907503). The registration date of the clinical trial is 17th July, 2013.

## Introduction

Osteoarthritis (OA) is a common, debilitating joint disease that can result in disability, especially in the elderly [1]. It usually presents with joint symptoms, including pain, stiffness, and structural pathology affecting the articular cartilage and surrounding tissues [2]. OA mainly occurs in the large joints of the lower extremities, such as hip and knee. Hip OA is a chronic intra-articular disease characterized by pain during activity, decreased mobility, and impaired body movement that greatly affects the daily life of middle-aged and elderly populations worldwide.

The presence of hip OA and the severity level can currently be assessed using several tools, including X-ray, computed tomography (CT), magnetic resonance imaging (MRI), ultrasonography (US), Kellgren-Lawrence (KL) scale, etc [3]. In clinical routine, X-ray is considered as the first imaging choice in major rheumatology textbooks due to its easy accessibility and cost-effectiveness, but its radiation exposure should not be overlooked [4]. A typical pelvic X-ray exposes the patient to approximately 0.6–1.1 mSv of radiation per exam, which, while considered low, still warrants caution, particularly for repeated imaging or use in younger or vulnerable populations. Known as the gold standard, the KL grading scale assigns a 5-level grading framework from 0 to 4 to diagnose hip OA based on X-ray findings [5]. Here 0 indicates an asymptomatic joint, while grades 1 to 4 describe the OA condition as suspicious, mild, moderate and severe, respectively [6]. However, the KL score represents a condensed scale and does not accurately map to various imaging features associated with OA [7]. Several recent studies have shown that important contrasts between patient symptoms and imaging findings can be observed. Patients with significant radiographic changes do not necessarily exhibit severe associated clinical symptoms, and vice versa. Specifically, patients with radiographically high-grade hip OA may be asymptomatic [8]. In cases where hip OA is suspected but not confirmed by radiographs, it is recommended to consider other possible causative factors and diagnostic tools to explain the patient’s symptoms and confirm the presence of hip OA.

As machine vision, motion capture and biomechanical techniques have advanced rapidly, biomechanical abnormalities are increasingly quantified by the harmless gait analysis in OA. Understanding the impact of OA on gait can expedite the diagnosis of the disease at its earliest stages, the planning of physical therapy and surgical treatment, and the assessment of the effects of these treatments [9]. Hip OA significantly impairs gait efficiency, thus gait parameters such as stride length, cadence and speed are altered in patients with hip OA compared with healthy controls (HCs) [10]. In addition to changes in kinematic gait parameters, patients have diminished range of motion (ROM) in three dimensions of hip, knee and ankle joints prior to total hip arthroplasty [11]. Variability in motion during gait can accelerate the progression of hip OA to varying degrees [12]. Additionally, according to several studies, hip OA patients with altered gait also experience abnormal loads in their lower extremities. There is evidence that physical and mechanical changes in one joint can affect all joints in the lower extremity with OA, based on biomechanical studies in the lower extremity [13]. Variability in lower extremity kinematic chain including hip, knee and ankle joints assesses different aspects of pathological kinematic variability [14] and may be associated with hip OA [11]. According to a traditional perspective on endpoint variability, patients with severe hip OA would have more variability in their lower extremity kinematic chains to prevent painful structural joints from being subjected to repetitive loads [15]. Endpoint variability can be assessed as gait variability and correlates with pain relief. Indirectly, a relationship between hip OA severity and lower extremity joint variability can be established [11, 16].

To quantify gait variability, several techniques have been proposed, such as gait variability index (GVI) [17], Kernel density estimation (KDE) [18], spectral arc length measure (SALM) [19], phase coordination index (PCI) [20], Tekscan analysis programs (TAP) [21], wavelet transform-based power spectral density (PSD) [22], etc. In addition, nonlinear analysis also plays an important role in quantifying gait variability and revealing its relationship with hip OA severity. Nonlinear metrics, such as multiscale entropy, recurrence quantification analysis (RQA) [23], fractal analysis, largest Lyapunov exponent (LLE) [24], and sample entropy [25], have been widely used to describe the pathological gait variability. In terms of entropy, several methods have been used to study time series complexity, such as sample entropy, approximate entropy, fuzzy entropy and permutation entropy [26]. In nonlinear signal analysis, Lempel-Ziv complexity (LZC) is an important metric to detect dynamic changes [27].

After quantifying gait variability, multiple techniques including statistical analysis, machine learning, and artificial intelligence were utilized to detect the presence of hip OA and grade the severity using quantified features. For example, statistical indices use Bonferroni correction from hip angle and moment in unilateral hip OA patients to investigate the association between radiographic severity and pain and loading changes [13]. Likewise, Liao et al. [28] investigated the correlation between 3-dimensional (3D) kinematic and kinetic hip parameters, patients’ symptoms, and cartilage morphology. Statistical analysis was performed to compare the difference between hip OA patients and HCs. Compared with HCs, radiographic hip OA patients had larger moments of hip abduction and adduction during walking, along with larger hip flexion, adduction, and external rotation moment impulses. In the research conducted by Teufl et al. [29], kinematic data were extracted from hip OA patients and 3D ROM of the hip was computed using an inertial sensor system. The impaired gait was distinguished from the unimpaired gait with the help of a support vector machine (SVM) model. Although previous studies have offered valuable research insights, the features they selected were relatively limited, failing to provide sufficient discriminative information, and did not further differentiate between different levels of disease severity.

Overall, the main contributions of our proposed model can be summarized as follows.

1. This study establishes a comprehensive framework for feature extraction and selection, successfully identifying several discriminative spatiotemporal and nonlinear features, which utilizes measurements from the sagittal, coronal, and transverse planes of the hip, knee, and ankle joints, providing a robust foundation for analyzing multidimensional kinematic data in gait studies.
2. This study applies the interpretability analysis for the feature selection and results prediction. By assigning feature importance values to each input variable, the Shapley Additive exPlanations (SHAP) method [30, 31] enhances the transparency and comprehensibility of the SVM. In this study, the SHAP value of the coefficient of variation of gait cycle ranked first across all classification tasks, significantly outperforming other features. Therefore, this feature can be considered for use in clinical diagnosis and grading.
3. This study demonstrates the ability to detect hip OA with a binary classification (HCs *vs* hip OA patients) accuracy of 98.21%. Additionally, the grading of hip OA is achieved with accuracies of 89.65% for the ternary classification (HCs *vs* Grade2/3 *vs* Grade 4) and 87.54% for the quaternary classification (HCs *vs* Grade2 *vs* Grade 3 *vs* Grade 4).

Throughout the article, the following structures are followed. The proposed method is depicted in detail in Method section, which includes description of the dataset used, extraction, and selection of spatiotemporal and nonlinear features, and classification model. Results section designs comprehensive experiments and provides corresponding results. Discussion section presents comprehensive discussion about results and contribution. Conclusion section gives a brief conclusion.

## Method

**Fig. 1:**
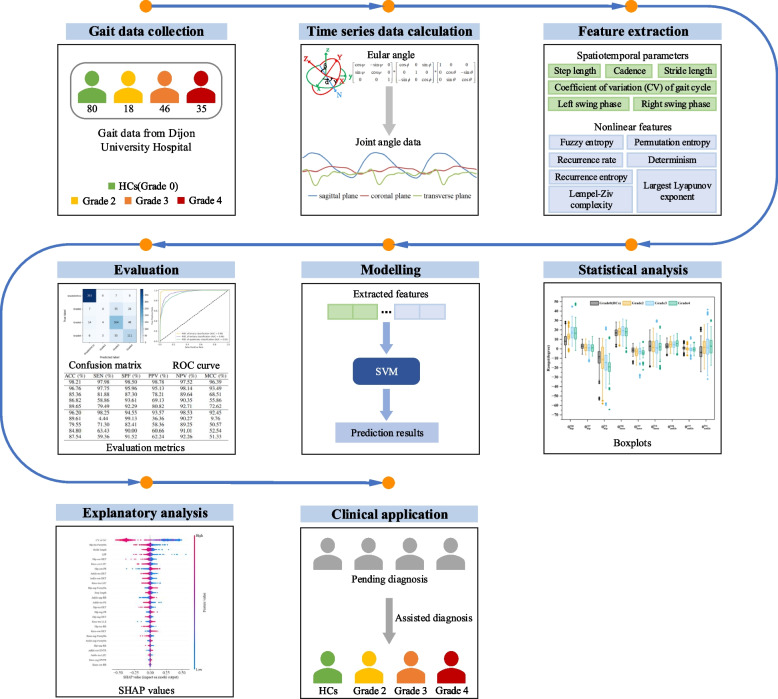
The workflow of the interpretable machine learning approach

A description of the proposed method for distinguishing hip OA from asymptomatic gait patterns follows in this section. Figure 1 illustrates how the proposed method is applied to binary and multi-class classification problems. It involves two main phases: feature extraction and classification. Each phase includes multiple steps to achieve the desired results. First, spatiotemporal and nonlinear features are extracted using different methods such as fuzzy entropy, permutation entropy, LZC, LLE and RQA. In the second step, SHAP is used for feature selection and dimensionality reduction. Afterwards, the classification performances among different tasks is compared by classifying the gait patterns of different groups using feature vectors and SVM. Finally, distinct performance parameters are utilized to evaluate the classification results.

### Dataset description

This study utilizes the hip OA dataset constructed by Bertaux et al. [32]. A study conducted by the University Hospital of Dijon (France) between 2011 and 2016 enrolled 80 healthy control subjects and 106 participants with unilateral hip OA who volunteered to participate. Out of the hip OA patients, four individuals did not furnish their KL grade while three individuals did not offer any data. Consequently, only 99 patients were included in our study. Table 1 illustrates the information about the participants’ demographics and anthropometrics. Here, Grade 1 data is missing in the dataset. The American College of Rheumatology criteria were applied to diagnose hip OA, including an evaluation of the radiographs. The criteria for exclusion included onset of OA, ankle pain, extremity disease, chronic or acute back pain, neuromuscular disease, Parkinson’s disease, heart or respiratory failure, diabetes, or a number of causes from clinical gait analysis. The protocol complied with Good Clinical Practice (ICH Harmonized Tripartite Guidelines, 1996) and the Declaration of Helsinki. Before participation, the study was conducted with informed consent from all participants, and its ethics committee approved it (CPP Est I, Dijon, France). NCT01907503 clinical trials have been cited on ClinicalTrials.gov [32].

Eight photoelectric cameras (Vicon MXT40, Vicon, UK) completed gait data collection at 100 Hz for this dataset. A plug-in gait model was used to position reflective skin markers on all participants. Each marker set was calibrated for each patient prior to ambulation, and static recordings were performed. An 6-meter-long horizontal walkway required participants to walk up and down it multiple times. The operator validated all trials quickly after recording at least five trials for each condition [32]. For detailed data collection procedure, please refer to Bertaux et al. [32].

**Table 1:** Demographic Information of HCs and hip OA patients

	HCs (Grade 0)	Grade 2	Grade 3	Grade 4
Age (years)	58.7 (15.5)	68.8 (8.77)	66.1 (10.0)	67.1 (9.32)
Height (m)	1.66 (0.09)	1.62 (0.08)	1.65 (0.09)	1.64 (0.09)
Weight (kg)	69.3 (13.4)	72.6 (16.0)	77.0 (16.4)	81.0 (17.3)
Male/Female	35/45	6/12	22/24	19/16

In conjunction with Vicon Nexus software (Nexus 2.10, Vicon, UK), marker trajectories were labelled [32]. After interpolating the trajectory with a Woltring spline algorithm, a low-pass Butterworth filter with a cutoff frequency of 10 Hz was used to smooth it. A plug-in gait model was used to calculate joint kinematics with Vicon Nexus.

### Feature extraction

This study discusses spatiotemporal parameters $F_S$ and nonlinear metrics $F_N$ for more efficient feature extraction. From sagittal, coronal, and transverse kinematic data in the hip, knee, and ankle joints, $F_N$ is calculated using multiple entropies, RQA, LZC, and LLE.

#### Spatiotemporal parameters

Six spatiotemporal parameters, including step length (m), cadence (step/s), stride length (m), coefficient of variation (CV) of gait cycle (GC), left swing phase (LSP) and right swing phase (RSP), are used to fully reflect the characteristics of gait movement between hip OA patients and HCs. Step length is the distance between the heels of one footprint and the heels of the opposite footprint in anteroposterior direction [33]. Cadence means steps per second or minute. In a walk, a stride length is measured by the distance between the heels of two consecutive footsteps (heels) [33]. The CV of the gait cycle can be considered as an indicator of gait stability, and it is calculated as $CV=\frac{GC_{std}}{GC_{mean}}$. Here, $GC_{std}$ represents the standard deviation (std) of GC, and $GC_{mean}$ represents the average value of GC. An LSP corresponds to the period in which the left foot swings forward in the air (without touching the ground), while an RSP corresponds to the period in which the right foot swings forward in the air. Here, we redefine LSP and RSP using percentages of gait cycles, which are calculated as follows. LSP($\%$)=$\frac{the~time~of~left~swing~pahse}{GC}$, RSP($\%$)=$\frac{the~time~of~right~swing~pahse}{GC}$. Therefore, the spatiotemporal features $F_S=\mathrm {[step~ length,~cadence,~stride~length,} \mathrm {~CV~of~GC,~LSP,~RSP]}^T$.

**Fig. 2:**
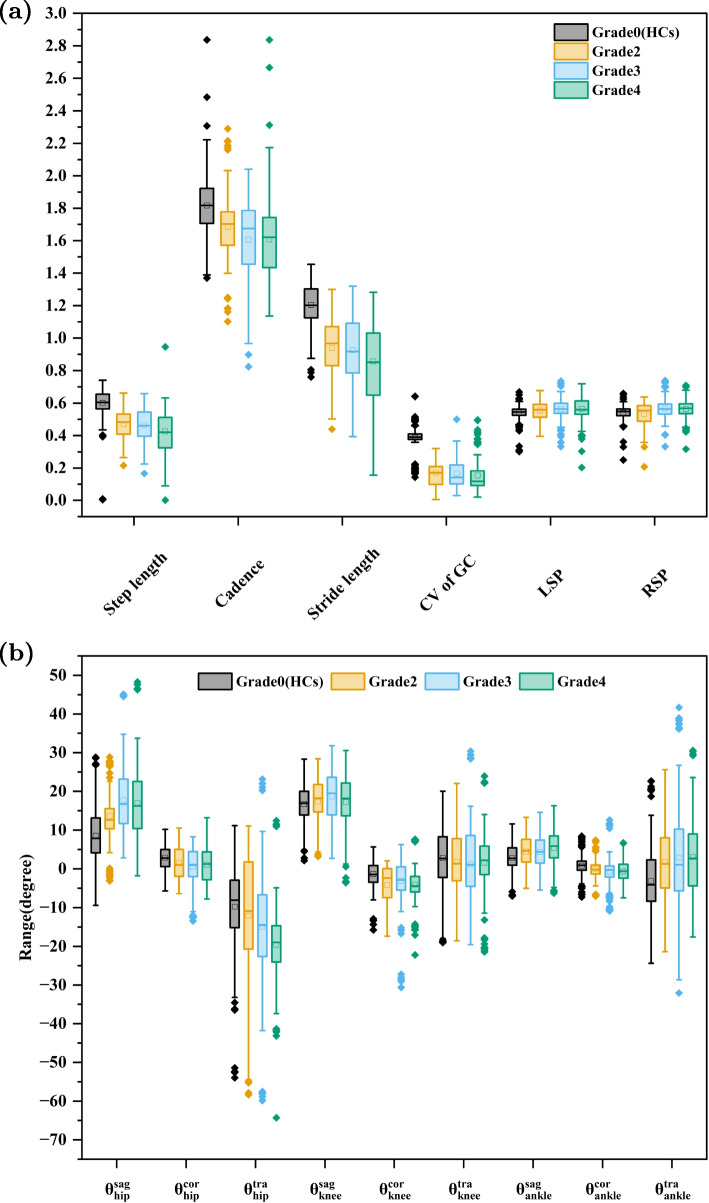
Boxplots for the (**a**) spatiotemporal parameters and (**b**) joint angles in three dimensions

As shown in Fig. 2, the boxplots depict the spatiotemporal parameters $F_S$ and joint angles in three dimensions. Here, $\theta _{hip}^{sag}$, $\theta _{hip}^{cor}$ and $\theta _{hip}^{tra}$ represent hip angles in three dimensions (the sagittal, coronal and transverse planes). $\theta _{knee}^{sag}$, $\theta _{knee}^{cor}$ and $\theta _{knee}^{tra}$ represent knee angles in three dimensions. $\theta _{ankle}^{sag}$, $\theta _{ankle}^{cor}$ and $\theta _{ankle}^{tra}$ represent ankle angles in three dimensions. These joint angles will be used for extracting the following nonlinear features. Boxplots are created based on the median, upper quartile, lower quartile, and whisker values. These plots display descriptive statistics of each feature, such as outliers, symmetry, and other gait details, in order to provide a clear representation of their distribution [34]. A distinction is made between spatiotemporal parameters and joint angles. The criterion for distinguishing among these features is based on the observation that a high concentration of sample points in the boxplots representation of the data indicates an obvious misalignment.

#### Nonlinear features

Nonlinear gait features refer to characteristics of walking patterns that capture the complexity, irregularity, or dynamic variability in human locomotion beyond what traditional linear metrics (e.g., average step length or cadence) can detect. Unlike linear features that summarize central tendencies, nonlinear features are particularly effective in characterizing subtle variations over time in gait signals, variations that may reflect neuromuscular control, compensation strategies, or biomechanical instability associated with osteoarthritis.

For example, the LLE quantifies the divergence of nearby trajectories in a reconstructed state space and is commonly used to measure gait stability. A higher LLE indicates less stable gait dynamics, which may correlate with disease severity. Similarly, Sample Entropy and (LZC measure the unpredictability and complexity of gait time series data, with higher values reflecting greater irregularity in joint movement patterns. These nonlinear features provide a more detailed view of functional impairment than traditional kinematic summaries.

Using features such as entropies, RQA, LZC, and LLE, we can measure the variability or irregularity of nonlinear time series. The representation and meaning of each nonlinear feature are presented in Table 2. To sum up, the nonlinear feature $F_N = [FuzzyEn, PE, RR, DET, ENTR, LZC, LLE]^T$.

All nonlinear features were extracted from the kinematic time series data of hip, knee, and ankle joint angles in the sagittal, coronal, and transverse planes. Prior to feature extraction, each time series underwent preprocessing including normalization to zero mean and unit variance, resampling to 100 data points per gait cycle, and application of a 4th-order Butterworth low-pass filter with a 10 Hz cutoff frequency.

The extraction of nonlinear features was implemented in Python 3.8 using specialized time series analysis libraries. For each metric, the following computational procedures and parameters were employed:

1. FuzzyEn: Computed using the PyEEG library (v0.4.4) with parameters m = 2 (embedding dimension), r = 0.2 $\times$ std (similarity threshold), and n = 2 (vector similarity fuzzy power). FuzzyEn measures the irregularity of time series data while being less sensitive to noise than sample entropy by employing a fuzzy membership function.
2. PE: Implemented using the Entropy package (v0.5.1) with parameters order = 3 (permutation order) and delay = 1 (time delay). PE quantifies the complexity of a time series by analyzing the permutation patterns of values, capturing the temporal dynamics of gait variability.
3. RQA metrics: Computed using the PyRQA package (v0.3.0) with embedding dimension = 3, time delay = 1, radius = 0.15 $\times$ std of the signal, and line length = 2. From the recurrence plots, we extracted:
  RR: Measures the density of recurrence points in the recurrence plot
  DET: Quantifies the proportion of recurrence points forming diagonal lines
  ENTR: Measures the complexity of the deterministic structure in the system
4. LZC: Implemented based on the algorithm described by Lempel and Ziv. The time series was first converted to a binary sequence using the median value as the threshold. LZC quantifies the rate of new pattern generation in the time series, providing a measure of randomness.
5. LLE: Calculated using the nolds package (v0.5.2) with parameters embed$\_$dim = 3, lag = 1, and min$\_$tsep = 10. LLE characterizes the rate of separation of infinitesimally close trajectories in phase space, serving as a measure of local dynamic stability during gait.

For each participant, the nonlinear features were computed for each gait cycle and then averaged across all cycles per trial to obtain representative values. This process was repeated for all nine joint angle dimensions (3 joints $\times$ 3 planes), resulting in 63 nonlinear features per participant (7 metrics $\times$ 9 joint angle dimensions).

These nonlinear features, combined with the spatiotemporal parameters described in Spatiotemporal parameters section, formed the comprehensive feature set used for subsequent feature selection and classification tasks. The computation of nonlinear features from kinematic data enhances the ability to detect subtle changes in gait patterns associated with different grades of hip OA, which may not be captured by conventional spatiotemporal parameters alone.

**Table 2:** Representations and meanings of the nonlinear features in this study

Representation	Meaning
FuzzyEn	Fuzzy entropy [35, 36]
PE	Permutation entropy [37]
RR	Recurrence rate [38] of RQA [39, 40]
DET	Determinism [38] of RQA
ENTR	Recurrence entropy [38] of RQA
LZC	Lempel-Ziv complexity [41]
LLE	Largest Lyapunov exponent [42]

### Feature selection

Feature selection and effective dimensionality reduction are crucial steps in machine learning models to improve classification accuracy and reduce computational complexity. SHAP values can play a significant role in this process by helping to identify the optimal combination of high-dimensional features. SHAP is a game-theoretic approach used to evaluate the importance of each feature in a machine learning classifier’s prediction outcome. By computing the SHAP values for each feature, an intuitive and interpretable feature importance ranking can be obtained, and the reasons behind the entire prediction result can be explained. This approach provides an effective tool to help people better understand the decision-making process of SVM and makes them easier to be accepted and used.

The process of feature selection using SHAP can be described as follows. First, SHAP values are calculated for each feature of the feature set using SVM. These values quantify the impact of each feature on the prediction made by SVM for a given instance. Next, the features are ranked according to their mean absolute SHAP values across the dataset. This ranking provides an initial assessment of the relative importance of each feature in the model’s decision-making process. Then, a subset of the top-ranked features is selected based on a predetermined criterion, such as a fixed number of features or a certain level of cumulative importance. The selected subset is used to train a new model, which is evaluated using a suitable performance metric. If the performance of the new model is satisfactory, the selected subset of features can be considered as the optimal feature set for the given task. Otherwise, the feature selection process may need to be repeated with different SHAP values, model architectures, or selection criteria until a satisfactory result is achieved. Overall, the SHAP-based feature selection approach provides a principled and interpretable way to identify the most relevant features for a given machine learning task, while taking into account their interactions and nonlinear effects.

### Classification model

SVM is a commonly used machine learning method for solving classification problems in nonlinear feature spaces [43], hence it is evaluated for a comparative study. Additionally, it is suitable for datasets of small size, as in this research.

For classification, we used a SVM with an RBF kernel, which is well-suited for nonlinear decision boundaries. Hyperparameters were tuned using a grid search approach nested within the 10-fold cross-validation framework to prevent data leakage. The search space included $C \in {0.1, 1, 10, 100}$ and $\gamma \in {0.001, 0.01, 0.1, 1}$. The optimal parameters selected based on cross-validation performance were $C = 10$ and $\gamma = 0.01$ across most classification tasks. These settings were used for all reported SVM results. This tuning strategy ensures that the reported performance reflects robust model selection without overfitting.

To evaluate the performance of our proposed approach, we implemented a subject-wise 10-fold cross-validation procedure. The dataset was partitioned at the participant level rather than at the trial level, ensuring that all gait trials from a single participant were assigned to the same fold. This methodological precaution prevents data leakage between the training and testing phases, as trials from the same subject often share subject-specific gait characteristics. The 179 participants (80 healthy controls and 99 hip OA patients) were randomly stratified into 10 folds while maintaining the approximate class distribution. For each fold iteration, approximately 90% of the participants’ data were used for training, and the remaining 10% were used for testing. The confusion matrices and performance metrics reported in our results represent the aggregated outcomes from the test sets across all 10 folds, providing a robust estimate of the model’s generalization ability to unseen participants.

**Fig. 3:**
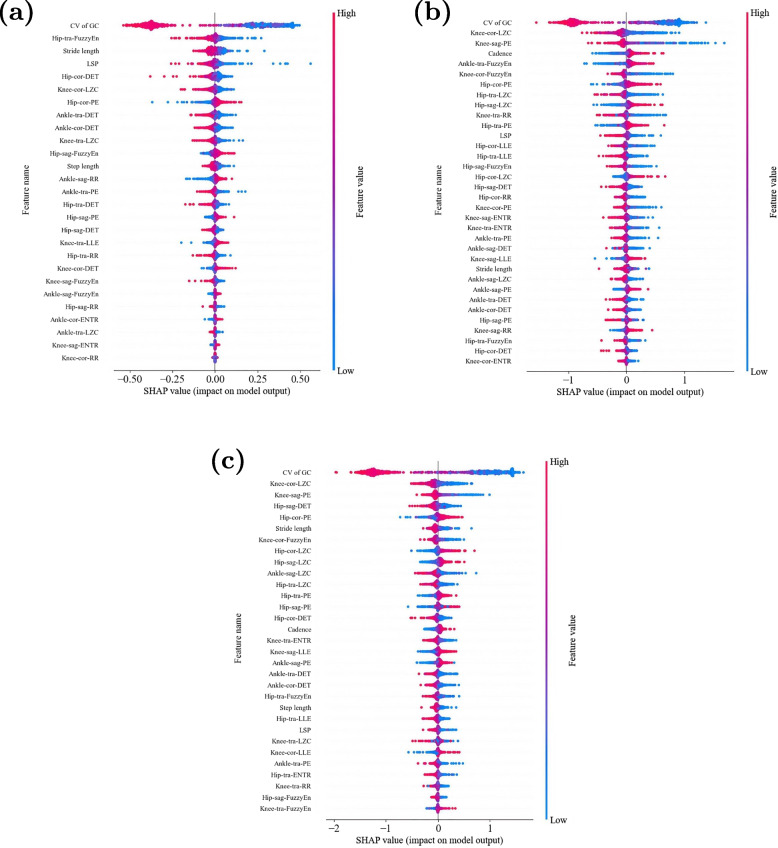
SHAP plots of the best feature sets in (**a**) binary, (**b**) tertiary, and (**c**) quaternary classification. The features in each figure are arranged from top to bottom in descending order of importance

## Results

### Model evaluation metrics

Experiments are conducted to test the effectiveness of the proposed features on SVM. In addition to accuracy (ACC), specificity (SPF), and sensitivity (SEN), we use another four classic performance indicators: negative predictive value (NPV), positive predictive value (PPV), F1 score, and Matthews correlation coefficient (MCC).

### Feature selection based on SHAP value

This section demonstrates the ranking of the importance of feature selection based on SHAP value for SVM. To identify the optimal combination of features, the SHAP values for each feature are computed and subsequently ranked according to their importance. Only the most significant features are retained, resulting in a reduction in the dimensionality of the feature space. For example, in our experiments, the total number of spatiotemporal and nonlinear features is $6+7\times 3\times 3=69$. These features are then used for model training, resulting in significant improvements in classification accuracy and a reduction in computational complexity. Figure 3 displays the SHAP summary plots of the top-performing feature sets utilized by the classifiers. This figure illustrates that the selected spatiotemporal and nonlinear metrics are all incorporated into these feature sets.

### Comparison of classification tasks

**Fig. 4:**
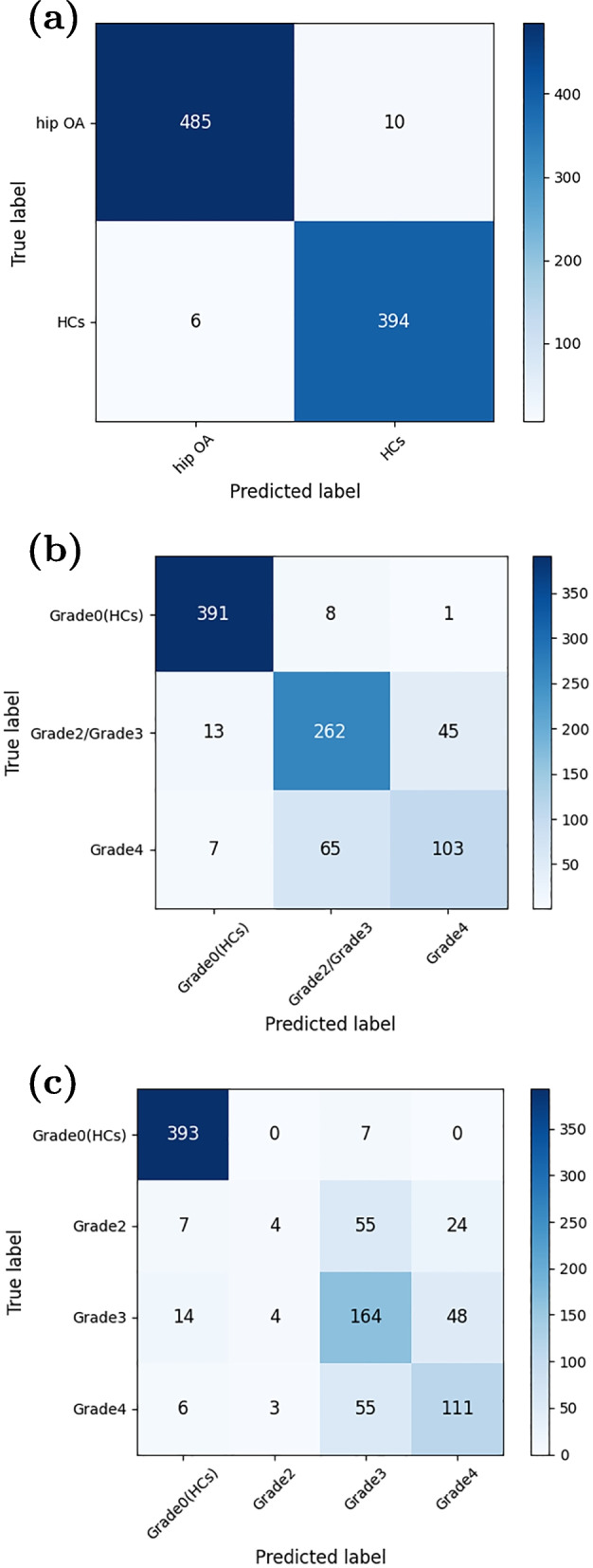
The confusion matrices of (**a**) binary, (**b**) tertiary, and (**c**) quaternary classification

The overall confusion matrices for SVM in binary, ternary, and quaternary classification tasks are obtained using a cross validation scheme of 10 folds. These matrices are presented in Fig. 4. The classification performance of SVM for the three types of tasks is summarized in Table 3.

**Table 3:** Performance indexes of three classification tasks for SVM using 10-fold cross-validation scheme

Task		ACC (%)	SEN (%)	SPF (%)	PPV (%)	NPV (%)	MCC (%)
Binary classification	/	98.21	97.98	98.50	98.78	97.52	96.39
Tertiary classification	Grade0 (HCs)	96.76	97.75	95.96	95.13	98.14	93.49
	Grade2/Grade3	85.36	81.88	87.30	78.21	89.64	68.51
	Grade4	86.82	58.86	93.61	69.13	90.35	55.86
	**Average**	89.65	79.49	92.29	80.82	92.71	72.62
Quaternary classification	Grade0 (HCs)	96.20	98.25	94.55	93.57	98.53	92.45
	Grade2	89.61	4.44	99.13	36.36	90.27	9.76
	Grade3	79.55	71.30	82.41	58.36	89.25	50.57
	Grade4	84.80	63.43	90.00	60.66	91.01	52.54
	**Average**	87.54	59.36	91.52	62.24	92.26	51.33

**Fig. 5:**
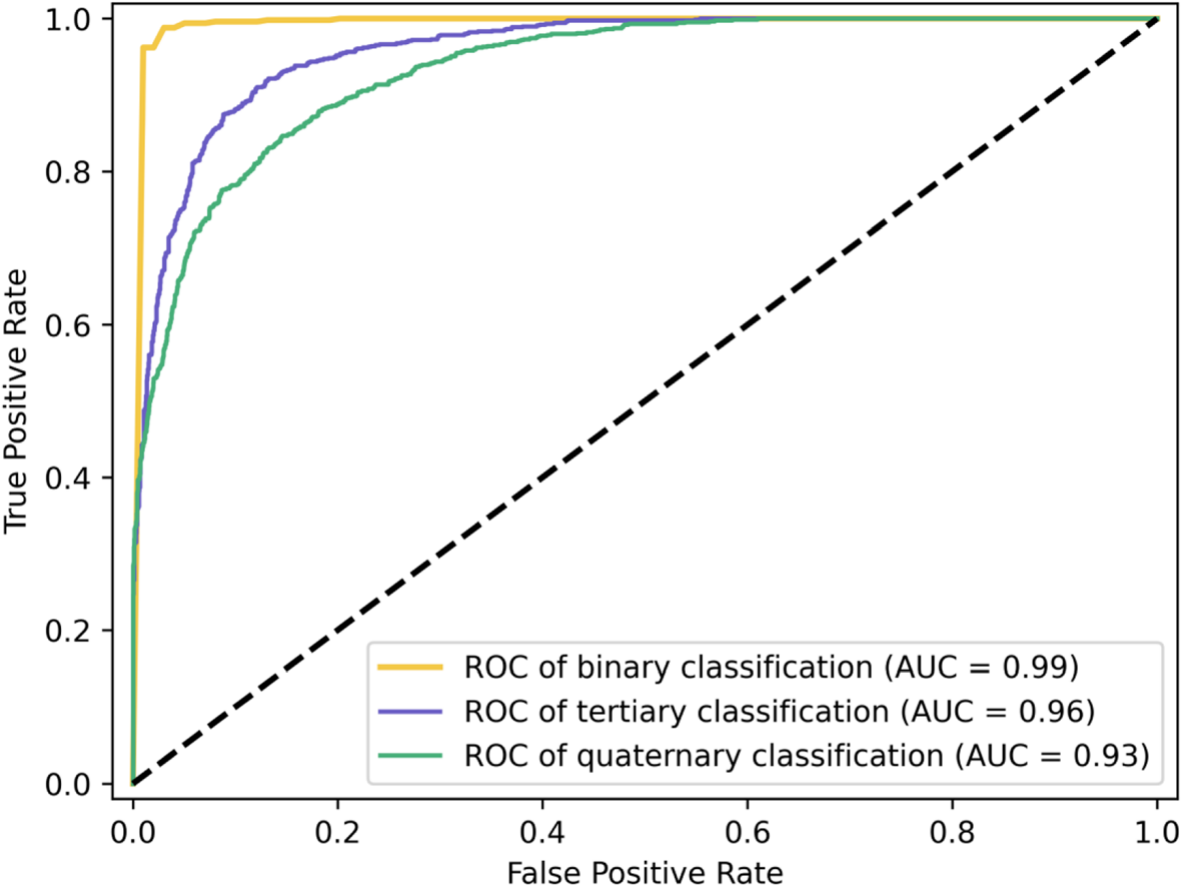
The comparison of the ROC curves of SVM on binary, ternary, and quaternary classification tasks

In Fig. 5, the receiver operating characteristic (ROC) curves illustrate how SVM performs in binary and multi-class classification, respectively. Area under the ROC curve (AUC) can be used to summarize classification performance. In this study, we apply a cross-validation scheme of 10-fold, for each repeated iteration, calculate the AUC, and summarize classification performance based on the average AUC. We achieve excellent results with our classification approach as a whole. The results suggest that the utilization of SVM in conjunction with spatiotemporal and nonlinear features can effectively detect hip OA and rate the corresponding severity level, thus indicating the success of our method in this regard.

**Table 4:** The results (mean values of the indicators) of the comparative experiments

Task	Model	ACC (%)	SEN (%)	SPF (%)	PPV (%)	NPV (%)	MCC(%)
Binary classification	RF	93.63	93.13	94.25	95.25	91.73	87.18
	LR	96.42	95.96	97.00	97.54	95.10	92.80
	LSTM	72.51	72.08	72.08	72.20	72.20	44.28
	CNN-LSTM	92.29	92.26	92.26	92.17	92.17	84.43
	Ours	98.21	97.98	98.50	98.78	97.52	96.39
Tertiary classification	RF	83.61	69.39	87.41	70.39	88.44	57.78
	LR	85.18	73.17	89.13	72.96	89.25	62.25
	LSTM	71.99	53.36	77.42	55.29	78.14	32.02
	CNN-LSTM	85.47	74.20	88.77	75.45	89.14	63.75
	Ours	89.65	79.49	92.29	80.82	92.71	72.62
Quaternary classification	RF	84.75	52.73	89.20	52.51	90.64	42.24
	LR	85.08	58.43	90.24	58.54	90.45	48.81
	LSTM	78.10	41.47	82.98	42.04	84.99	25.73
	CNN-LSTM	85.31	56.03	89.69	55.56	90.45	45.80
	Ours	87.54	59.36	91.52	62.24	92.26	51.33

### Comparative experiments

Recent advances in deep learning have led to its widespread adoption in time-series motion analysis, including applications in musculoskeletal modeling and hip OA classification. Recurrent neural networks (RNNs), particularly Long Short-Term Memory (LSTM) models, have demonstrated strong performance in capturing the temporal dynamics of biomechanical signals. For instance, Perrone et al. [44] developed LSTM models for accurate prediction of hip joint moments from wearable data. Similarly, Pantonial et al. [45] proposed a hybrid CNN-LSTM model for hip OA detection, reporting high accuracy while preserving spatial-temporal dependencies in gait cycles. Meanwhile, we also conducted experiments on traditional machine learning models, such as Random Forest (RF) and LR, to obtain more comprehensive comparative results. The experimental results are shown in Table 4.

It is evident that the SVM used in this study achieved the best performance across all three classification tasks. Compared to the other four classification models, SVM demonstrated stronger competitiveness in classifying this dataset.

## Discussion

### Comparison of methods

Experimental results from this study suggest that gait analysis can detect and assess hip OA severity automatically based on spatiotemporal and nonlinear features. In addition to providing a comprehensive machine learning pipeline that can handle discriminative gait covariates, the proposed scheme provides a supplementary tool to the KL scale as well for presence detection and severity rating of hip OA in an automatic and objective manner. The classification performance comparison between our method and other up-to-date algorithms is illustrated in Table 5.

**Table 5:** Comparative experiments for the classification of hip OA patients and HCs

Method	No of participants	Features	Classifier	2-class	3-class	4-class
Comparison of different studies (image data)						
Xue et al. (2017) [46]	201 (hip OA)+219(HCs)	X-ray images	CNN	92.8	N/A	N/A
Üreten et al. (2020) [47]	213 (hip OA)+221 (HCs)	Hip radiographs	VGG-16	90.2	N/A	N/A
Yamamoto et al. (2020) [48]	598 (hip OA)+535 (HCs)	Hip radiographs and clinical covariates	EfficientNet b3	88.5	N/A	N/A
Boniatis et al. (2007) [3]	46 (hip OA)+18 (HCs)	Shape features from HJS images	DT	98.4	96.9	N/A
Comparison of different methods on the dataset this study uses (gait data)						
Laroche et al. (2014) [49]	99 (hip OA)+80 (HCs)	Kinematic gait trajectories	SVM	71.34	69.28	75.58
Emmerzaal et al. (2022) [50]	99 (hip OA)+80 (HCs)	Lumbar spine and hip kinematics	LR	88.80	79.77	81.24
Proposed method (ours)	99 (hip OA)+80 (HCs)	Nonlinear features + spatiotemporal parameters	SVM	98.21	89.65	87.54

Laroche et al. [49] used a 3D motion capture system to extract motion trajectory data, including spatial angles, torso and pelvis motions in three planes. Afterwards, the SVM classifier was applied to the kinematic data to classify patients with hip OA and HCs with 90% accuracy. Additionally, they examined the correlation of kinematic data with Western Ontario and McMaster University (WOMAC) severity scores.

Using a variety of statistical models with varying levels of data complexity, Emmerzaal et al. [50] sought to differentiate hip OA patients from healthy control subjects. The study involved 20 patients with hip OA and 12 HCs. The first class covered kinematics only, the second covered joint dynamics, and the third covered musculoskeletal modeling data. Machine learning models were used to evaluate classification performance. They found that the first class using the LR model with seven kinematics features of lumbar spine and hip achieved the best accuracy of 93.9% when climbing stairs.

Xue et al. [46] evaluates the classification performance of deep convolutional neural networks (CNNs) on hip X-ray images from 201 OA samples and 219 normal samples. According to their report, the highest accuracy, sensitivity, and specificity were 92.8%, 95.0%, and 90.7%, respectively.

Üreten et al. [47] investigated the impact of plain pelvic radiography on the diagnosis of hip OA. A pretrained VGG-16 network was used to model a total of 221 radiographs of normal hips and 213 radiographs of hip OA. A score of 90.2% was found in accuracy, 97.6% in sensitivity, 83.0% in specificity, and 84.7% in precision.

To classify hip OA, Yamamoto et al. [48] used hip radiography, clinical covariates, and deep learning models. The evaluation involved 598 hip OA radiographs and 535 non-OA radiographs. Patient clinical covariates included age, BMI, gender, and hip fracture history. With fine-tuning and ensemble learning, five CNN models were evaluated for modality-specific transfer learning, and Efficient Net b3 achieved the highest accuracy of 88.5%.

Currently, most existing studies focus on differentiating patients with hip OA from HCs, which is a binary classification case. In contrast, there have been very few literature reports on multi-class classification dealing with severity rating issues. Boniatis et al. [3] extracted shape features from hip joint space (HJS) images and fed them into a hierarchical decision tree for hip OA detection and severity rating. Participants included 18 HCs, 16 patients with grade 2/3 (mild/moderate) hip OA, and 30 patients with grade 4 (severe) hip OA. The best reported accuracy is 98.4$\%$ for binary classification and 96.9$\%$ for ternary classification (Grade 0 versus Grade 2/Grade 3 versus Grade 4), respectively. However, a small number of participants limited the experiment to one type of multi-class classification, which was insufficiently convincing.

As far as we know, this is the first study to use kinematic gait data in comparison with conventional imaging methods to determine hip OA severity. In Table 4, the proposed method is demonstrated to be superior to other state-of-the-art methods. It indicates that as an auxiliary technique, gait analysis can be used for the diagnosis of hip OA as well as for grading severity. It is simple, objective, non-trauma and easily accessible, and has the potential to detect the presence and rating the severity level of hip OA.

### Interpretability analysis

SHAP value is utilized in this study for feature selection and dimensionality reduction. It is a metric used to evaluate the importance of features in a dataset, which measures the degree to which each feature contributes to the predictive power of a model. The relationship between SAHP value and summary plots is that they both aim to identify the most important features for a given problem.

As depicted in Fig. 3, the features with high SHAP values in the best feature sets for multi-class classification tasks are mainly focused on the hip and knee joints. Referring to the previous studies, the degenerative changes in the hip joints can be compensated by enhancing pelvic motion and muscle activation, or by adjusting movements in other joints of the lower limbs [51, 52]. Meanwhile, in the three classification tasks, the CV of GC consistently ranked first in SHAP values, demonstrating significantly higher importance than other features. This indicates that there are notable differences in gait cycle consistency and stability among subjects of different categories, making it easier for the model to capture these distinctions. Existing researches have shown a strong correlation between the spatiotemporal parameters of gait and the severity of hip osteoarthritis [53]. Therefore, the CV of GC is expected to become a clinical indicator for quantifying abnormal variations in spatiotemporal parameters, enabling the differentiation of subjects with varying degrees of severity.

### Deep learning in gait-based hip OA classification

While our current study adopts a feature-based SVM approach, motivated by the relatively limited sample size and the requirement for model interpretability, we recognize that transforming raw time series into handcrafted features may reduce the temporal richness of the data. Therefore, future work will involve training and comparing deep learning models that operate directly on joint angle sequences or full-body kinematic trajectories. These approaches may further enhance classification performance and enable more scalable, end-to-end clinical applications.

### Integration into clinical workflows and decision-making

While the KL grading scale remains the clinical standard for radiographic assessment of hip OA, it is well known that structural changes observed on X-ray do not always align with symptom severity or functional impairment. In this context, gait analysis, particularly when enhanced by interpretable machine learning, offers a valuable complementary perspective.

Compared to radiography, gait analysis provides non-invasive, objective, and radiation-free functional information, making it suitable for longitudinal tracking of disease progression and treatment outcomes. Although traditional motion capture systems may have higher equipment costs and limited availability in primary care, recent technological advances in wearable inertial measurement units (IMUs) and mobile gait assessment platforms have significantly improved accessibility and reduced operational barriers. These systems enable clinicians to collect meaningful gait data in outpatient or rehabilitation settings without requiring imaging infrastructure.

Clinically, gait-based assessment can be particularly useful in the following scenarios: 1) Functional triage for patients presenting with pain or mobility issues but without clear radiographic evidence of hip OA. 2) Post-treatment monitoring, including after total hip arthroplasty or during physiotherapy. 3) Remote or telemedicine applications, where imaging access is limited but functional assessment remains essential. 4) Patient engagement and education, by visualizing and tracking gait changes over time.

Therefore, while we do not claim that gait analysis can fully replace the KL grading scale, we suggest that it holds significant complementary value, particularly in cases where functional assessment is prioritized or frequent imaging is impractical. Furthermore, the explainability provided by SHAP-based feature importance supports clinical trust and transparency, which are critical for adoption in real-world settings.

In more detail, our proposed model offers a non-invasive, objective, and interpretable framework for OA detection and grading based on gait kinematics. This enables several clinical integration pathways:

1. Outpatient Screening and Functional Triage: Gait data can be captured during routine clinical visits using wearable sensors or marker-based motion capture systems. Our model can immediately classify disease severity, supporting early identification of individuals with suspected OA, even in cases where radiographic evidence is equivocal or minimal.
2. Diagnostic Support Tool: The predicted grades, derived from biomechanical data, can complement radiographic findings and symptom assessments. This is particularly useful in cases where imaging and clinical symptoms are discordant. The model’s interpretable outputs (e.g., SHAP-based feature contributions) can be used by clinicians to justify decisions or prioritize further testing.
3. Longitudinal Monitoring and Therapy Evaluation: Because gait parameters are sensitive to biomechanical changes, the model can be used to monitor disease progression or response to interventions (e.g., physical therapy, pharmacologic treatment, or joint replacement surgery) over time.
4. Remote and Home-Based Assessment: With the proliferation of portable IMUs and smartphone-based gait apps, patients could record gait data at home. The model can be deployed remotely to assess changes and alert providers when functional deterioration is detected.
5. Integration with Electronic Health Records (EHR): The model can be embedded as a decision-support module within EHR systems, offering clinicians a real-time, quantitative indicator of hip OA severity alongside imaging and PROMs. The inclusion of interpretable metrics enhances transparency and supports shared decision-making.

In summary, the model provides a framework that is compatible with clinical workflows focused on early detection, functional monitoring, and patient-centered care. Its strengths in non-invasiveness, scalability, and interpretability offer a compelling adjunct to radiographic KL grading and support its integration into both diagnostic and longitudinal care pathways.

### Limitations and future work

#### Dataset

A significant limitation of our study is the poor performance in classifying early-stage hip OA (KL Grade 2), as evidenced by the extremely low sensitivity (4.44%) in the quaternary classification task. This underperformance can be attributed to several factors. First, our dataset contains a relatively small number of Grade 2 participants (n=18) compared to other classes, creating a substantial class imbalance that challenges effective model training. Second, the biomechanical manifestations of early-stage hip OA may be too subtle to be reliably captured by our current feature set, particularly when attempting to distinguish between multiple disease stages simultaneously.

This limitation has important clinical implications, as early detection of hip OA represents a critical opportunity for intervention before significant joint damage occurs. Our findings suggest that while gait analysis shows promise for distinguishing between healthy individuals and those with moderate-to-severe hip OA (KL Grades 3–4), its current utility for early-stage detection remains limited when using the features and classification approach described in this study.

Future research should focus on developing more sensitive features specifically targeting the subtle biomechanical alterations that occur in early-stage hip OA. This might include more sophisticated analysis of movement variability, joint coordination patterns, or compensatory mechanisms. Additionally, larger datasets with better representation of early-stage OA cases are essential for improving model performance for this critical subgroup. Alternative approaches might also consider multimodal assessments combining gait analysis with other clinical measures or imaging techniques to enhance early detection capabilities.

#### External validation

Another limitation of this study is the reliance on internal cross-validation without external validation on an independent cohort. While our subject-wise 10-fold cross-validation methodology provides protection against data leakage and offers a reasonable estimate of model performance, it cannot fully substitute for validation on data collected from different centers, equipment configurations, or patient populations. The absence of external validation limits our ability to definitively assess the generalizability of the proposed model to diverse clinical settings.

External validation is particularly important in gait analysis research due to the potential variability introduced by different motion capture systems, marker placement protocols, laboratory environments, and data processing methods. Additionally, demographic factors, cultural differences in movement patterns, and variations in clinical practice for osteoarthritis diagnosis and grading could impact model performance when applied in new settings.

To address this limitation, future work should prioritize multi-center collaborative efforts to validate and refine the proposed approach across diverse cohorts. This would involve:

1. Collecting validation data from multiple clinical centers with varied patient demographics and disease characteristics
2. Developing standardized protocols for gait data collection and processing to minimize methodological variability
3. Establishing calibration and harmonization methods to account for systematic differences between data collection systems
4. Investigating the model’s performance across different subpopulations to identify potential demographic or clinical factors that affect prediction accuracy

Such external validation efforts would provide more robust evidence regarding the generalizability of our approach and strengthen its potential for clinical implementation. Until such validation is performed, the performance metrics reported in this study should be interpreted as promising preliminary results that require further confirmation in diverse settings.

#### Patient-reported outcome measures (PROMs)

A key limitation of the present study is the absence of PROMs in the dataset, which restricts our ability to evaluate the clinical congruence between the predicted OA severity and the patient’s subjective experience. PROMs, such as pain intensity, functional limitation scores, and health-related quality of life, are essential for contextualizing objective assessments and ensuring that prediction outputs reflect clinically meaningful outcomes. Future research will aim to incorporate PROMs alongside biomechanical data to explore the correlation between gait-derived features and self-reported symptom burden. Such integration would help validate the model’s utility in patient-centered care and strengthen its clinical interpretability and adoption.

## Conclusion

This study utilizes gait-based spatiotemporal features and nonlinear features to detect and evaluate the severity of hip OA, employing feature extraction and selection techniques as well as machine learning models. To achieve this goal, we propose a computational pipeline of gait signals extracted from a motion capture system to assess motor symptoms of hip OA in the lower extremities. SHAP value method provides a powerful tool for feature selection, feature extraction, and dimensionality reduction, which can help us to build more efficient, interpretable, and accurate machine learning classifiers.

Our results demonstrate that gait analysis can extract discriminant information for the detection and severity rating of hip OA. In addition, nonlinear features reveal the non-stationary characteristics of gait motion and could be used to quantify gait variability between different severity levels of hip OA patients and HCs. It also highlights the experimental protocol’s potential for future investigation on gait variability pre- and post- hip arthroplasty and for assessing recovery performance. Further KL grading experiments are necessary in hip OA patients after arthroplasty to compare the changes in severity to determine the effectiveness of the surgery.

While our current study adopts a feature-based SVM approach, motivated by the relatively limited sample size and the requirement for model interpretability, we recognize that transforming raw time series into handcrafted features may reduce the temporal richness of the data. Therefore, future work will involve training and comparing deep learning models that operate directly on joint angle sequences or full-body kinematic trajectories. These approaches may further enhance classification performance and enable more scalable, end-to-end clinical applications.

## Data Availability

The dataset used and analysed during the current study is available from the corresponding author on reasonable request.
